# Synthesis and Insecticidal Activity of Fire Ant Venom Alkaloid-Based 2-Methyl-6-alkyl-Δ^1,6^-piperideines

**DOI:** 10.3390/molecules27031107

**Published:** 2022-02-07

**Authors:** Xiaoqing Wu, Guangyu Wang, Guangxin Xu, Li Chen

**Affiliations:** 1 State Key Laboratory of Integrated Management of Pest Insects and Rodents, Institute of Zoology, Chinese Academy of Sciences, Beijing 100101, China; wuxiaoqing@nibs.ac.cn; 2 School of Life Sciences, Institute of Life Science and Green Development, Hebei University, Baoding 071002, China; 17863800784@163.com (G.W.); jainxuguang@163.com (G.X.)

**Keywords:** piperideine, venom, *Solenopsis invicta*, biological activity, contact toxicity, injection toxicity

## Abstract

2,6-dialkylpiperideines found in the venom of *Solenopsis* (Hymenoptera, Formicidae) fire ants are a range of compounds possessing various biological activities. A series of racemic 2-methyl-6-alkyl-Δ^1,6^-piperideines were synthesized for chemical confirmation of the natural products found in fire ant venom, and the evaluation of their biological activity. Synthetic Δ^1,6^-piperideines and the natural compounds in the *cis*-alkaloid fraction of *Solenopsis invicta* had identical mass spectra and retention times. Their insecticidal activities against the third-instar larvae of cotton bollworm (*Helicoverpa armigera*) were evaluated by using injection and topical application methods. All three compounds exhibited no lethal effect at concentrations of 0.05–0.4 mol/L by topical treatment, but moderate lethal effect at 0.4 mol/L through injection treatment. Compound **6a** showed significantly higher activity than the natural insecticide nicotine. The differences in activity among compounds **6b**, **6c** and nicotine were not significant. The elongation of the carbon chain at the 6-position of the piperideine ring appears to decrease insecticidal activity.

## 1. Introduction

The red imported fire ant, *Solenopsis invicta*, known as one of the most notorious invasive species in the world [1], has caused serious negative impacts on public health, agriculture and ecosystems [2,3,4,5,6]. It often becomes the dominant ant species in infested areas outside of its native range, due to its aggressive foraging behavior and high reproductive capability [7]. Having powerful venom may account for its aggressiveness and competitive advantage. For the purpose of understanding its taxonomic and toxicological properties, the chemistry of fire ant venom has been well studied [8,9]. Characteristic alkaloids are the major components of fire ant venom, while proteins make up only a small proportion [10]. The alkaloid component represented by the solenopsins contains predominately 2-methyl-6-alkyl or 2-methyl-6-alkenyl piperidines [10,11,12,13], with a series of minor piperideine alkaloids and pyridine alkaloids [14,15,16,17]. Those piperidine alkaloids have been demonstrated to possess diverse biological activities, including necrotic, hemolytic, antimicrobial and insecticidal activities [18,19,20,21]. Additionally, piperidine alkaloids can induce several different physiological activities in mammals [22,23,24,25,26,27] and serve as semiochemicals in insect communication [28,29,30].

The piperideine alkaloid from fire ant venom was first discovered in *Solenopsis xyloni* [10]. A mixture of 2-methyl-6-*n*-undecyl-Δ^1,2^-piperideine and 2-methyl-6-*n*-undecyl-Δ^1,6^-piperideine, obtained by the dehydration of *cis*-2-methyl-6-*n*-undecyl-piperidine with *t*-butylhypochlorite, has been used to establish the chemical identity of an odd peak found in *Solenopsis xyloni*. Reduction of this mixture with deuterated NaBH_4_ results in the disappearance of both peaks, and the presence of both *cis*- (major) and *trans*-2-methyl-6-*n*-undecyl-piperidine (minor). The reaction performed on the *S. xyloni* venom using NaBD_4_ gave the same results, which confirms the identity of 2-methyl-6-*n*-undecyl-Δ^1,2^-piperideine [10]. Subsequently, series of Δ^1,2^- and Δ^1,6^-piperideine alkaloids from the workers and alate queens of the red imported fire ants have been identified on the basis of their characteristic mass spectra [14,31,32,33]. However, to date, chemical confirmation of these piperideines with synthetic standards has not been reported.

2,6-dialkylpiperideines were proposed to function as precursors of the corresponding 2,6-dialkylpiperidines in their biosynthetic pathways [14,34,35]. Over the past decades, the antimicrobial and insecticidal properties of piperideine alkaloids have been reported by several groups [36,37,38,39,40,41]. 2,6-dialkylpiperideines have been demonstrated as a class of compounds that may exhibit interesting biological activities. However, piperideine alkaloid has remained a poorly studied topic compared to piperidine alkaloid. In the present study, we synthesized a series of racemic 2-methyl-6-alkyl-Δ^1,6^-piperideines and evaluated their insecticidal activity. The presence of 2-methyl-6-alkyl-Δ^1,6^-piperideines was confirmed with synthetic samples as well.

## 2. Results and Discussion

### 2.1. Chemistry

**Scheme 1:**
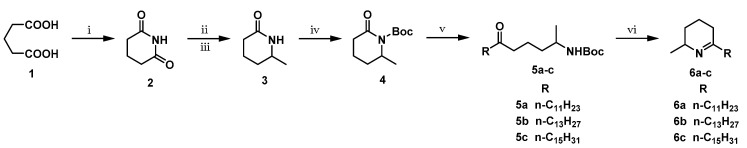
Synthesis of 2-methyl-6-alkyl-Δ^1,6^-piperideines. Reagents and conditions: (**ⅰ**) urea, neat, 175 °C, 2.5 h; (**ⅱ**) CH_3_MgBr/CH_2_Cl_2_, −78~25 °C, 12 h; (**ⅲ**) NaBH_3_CN, pH 3~4, r.t, 5 h; (**ⅳ**) (Boc)_2_O, Et_3_N, DMAP, CH_2_Cl_2_, reflux, 18 h; (**ⅴ**) RMgBr, TMEDA, THF, −30~25 °C, 12 h; (**ⅵ**) 12M HCl, CH_2_Cl_2_, 0 °C, 5 h.

Racemic 2-methyl-6-alkyl-Δ^1,6^-piperideines were prepared following the synthetic route shown in Scheme 1 [39]. The large-scale preparation of glutarimide (**2**) was readily carried out using glutaric acid (**1**) and urea as starting materials, which reacted neat at 175 °C. One of the carbonyl groups on the piperidinone ring was converted to a methyl group in compound **3** by a Grignard reaction of glutarimide (**2**) with CH_3_MgBr reagent, and subsequent reduction with NaBH_3_CN. A key step was the generation of NBoc-aminoketones **5a**–**c** from Boc-protected compound **4**, which was reacted with pre-prepared Grignard reagents to cause ring opening. Finally, the target compounds **6a**–**c** were obtained by catalytic cyclization of compounds **5a**–**c** with concentrated HCl. All copies of the spectra for compounds **2**–**4**, **5a**–**c**, and **6a**–**c** are available in Supplementary Materials.

### 2.2. Confirmation of Δ^1,6^-Piperideines in Natural Venom

**Figure 1:**
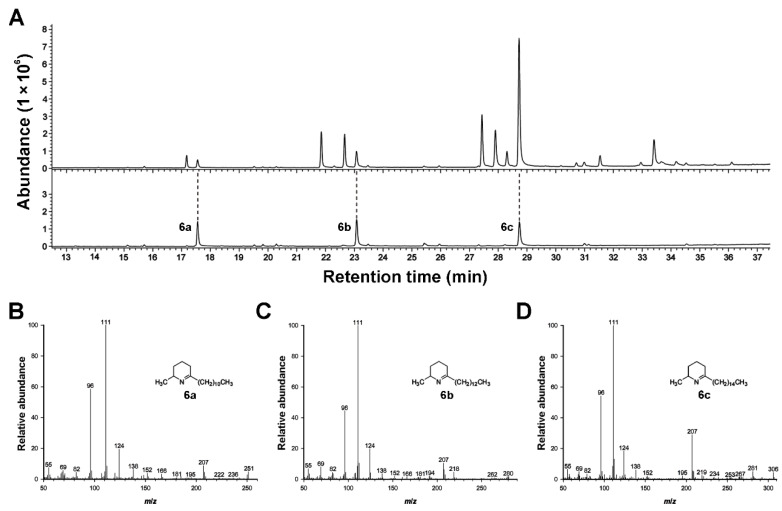
Chemical confirmation of Δ^1,6^-piperideines. (**A**) GC-MS chromatographs of the *cis*-alkaloid fraction and a mixture of compounds **6a**–**c**. (**B**–**D**) Mass spectra of compounds **6a**–**c**.

Fire ant venom alkaloids were readily extracted out of worker bodies and separated into *cis-* and *trans-*alkaloid fractions. The chemical identities of these alkaloids were determined by comparing the mass spectrum and the gas chromatographic retention time with published data. In both alkaloid fractions, two series of piperideines were isolated. Δ^1,6^-piperideines were identified by characteristic mass ions at *m*/*z* 96 and 111, whereas Δ^1,2^-piperideines were identified by characteristic mass ions at *m*/*z* 96, 97 and 110 [15,16]. Ultimately, synthetic compounds were directly compared with the natural alkaloids in the *cis*-alkaloid fraction, which have a well-established structure and stereochemistry. The retention times of Δ^1,6^-piperideine alkaloids from the natural venom are shown in Figure 1A. The mass spectra and retention times of synthetic compounds **6a**–**c** were completely consistent with those of the corresponding natural Δ^1,6^-piperideines. All three compounds had similar fragmentation patterns in the mass spectrum, with the intense ions at *m*/*z* 96 and 111 (Figure 1B–D). These data support the identification of Δ^1,6^-piperideine alkaloids in the venom of *Solenopsis* fire ants. The mass spectra of compounds **6a**–**c** matched those of the corresponding 2-methyl-6-undecyl-Δ^1,6^-piperideine, 2-methyl-6-tridecyl-Δ^1,6^-piperideine and 2-methyl-6-pentadecyl-Δ^1,6^-piperideine in the literature [10,14,15]. All these mass spectra showed the same base peak ion at *m*/*z* 111. However, Chen et al. [14] reported a slightly different mass pattern, with a base peak ion at *m*/*z* 96 for 2-methyl-6-tridecyl-Δ^1,6^-piperideine.

### 2.3. Toxicity against Cotton Bollworm (*Helicoverpa armigera*)

The insecticidal activities of compounds **6a**–**c** were evaluated by topical and injection treatments. As shown in Table 1, the mortality of piperideine **6b** and **6c** at various concentrations (0.05–0.4 mol/L) was similar to the standard control (nicotine) and the solvent control (acetone), indicating that these alkaloids were inactive against the early third-instar larvae of cotton bollworm at these test concentrations. Compound **6a**, with a shorter side chain, exhibited a weak effect within 48 h, through topical application at concentrations of 0.2 and 0.4 mol/L.

For the injection treatment, a concentration of 0.4 mol/L was selected. The three piperideines exhibited moderate activities against the third-instar larvae of cotton bollworm (Table 2). Compound **6a,** with an undecyl side chain, displayed the highest mortality rates of 45.5% (24 h) and 75.6% (48 h). Compounds **6b** and **6c,** with a longer side chain, showed relatively lower activity. The order of mortality from high to low is as follows: **6a** > **6b** > **6c**, which means that the piperideines with a shorter side chain have better activity. Interestingly, as shown in Table 2, **6a** displayed significantly higher activity in 48 h than nicotine, which has long been regarded as a natural insecticide [42]. At the same concentration, the toxicities of the piperideines in the subcutaneous injection were evidently greater than those in the topical administration, suggesting that the piperideines may possess weak penetrating capacity through the cuticle of the cotton bollworm larva. The significant difference in toxicity between topical and injection applications may explain why fire ant workers usually inject venomous poison into their prey while hunting.

The alkaloids in fire ant venom are mainly hydrophobic piperidines, along with piperideines in much lesser amounts. Piperideines have long been thought to be unstable intermediates for the biosynthesis of piperidines [10,15,35], but these remain largely unstudied. The facile syntheses of these Δ^1,6^-piperideines make it possible to study the biosynthesis of piperidines in fire ant venom. The mixed piperideine alkaloids from the extracts of the red imported fire ants show significant inhibitory effects on the growth of *Pythium ultimum* [36] and *Clavibacter michiganensis* subsp. *michiganensis* [41], and relatively high toxicity to the green peach aphid, *Myzus persicae* [38]. As they are minor components in fire ant venom, the availability of natural Δ^1,6^-piperideines is very limited. The synthetic version of Δ^1,6^-piperideines has the potential to control plant disease and pest insects. Future studies should focus on the scale-up synthesis of Δ^1,6^-piperideines and the development of Δ^1,6^-piperideine-based pesticides.

## 3. Materials and Methods

### 3.1. Synthesis

All reactions were carried out in dry glassware with a magnetic stirrer. Liquid solutions were transferred via syringe. All reactions were monitored by thin-layer chromatography on silica gel GF254 pre-coated plates (0.25 mm). The Grignard reagents *n*-C_11_H_23_MgBr, *n*-C_13_H_27_MgBr and *n*-C_15_H_31_MgBr were prepared according to a reported method from the corresponding *n*-alkyl bromide [43]. ^1^H and ^13^C NMR spectra were recorded on Varian Inova—400 MHz spectrometers using tetramethylsilane as the internal standard, and were reported in terms of chemical shift (δ ppm). The high-resolution mass spectra data were obtained using a Bruker mass spectrometer (Daltonik, Germany). Anhydrous THF was prepared by distillation over sodium with a diphenyl ketone indicator. Anhydrous DCM was collected by heating over CaH_2_. All other commercially obtained reagents and solvents were used as received.

Glutarimide (**2**): A mixture of glutaric acid (53.15 g, 0.40 mol) and urea (36.24 g, 0.60 mol) was melted for 40 min at 140 °C, and then the reaction mixture was stirred for an additional 2.5 h at 175 °C. Upon cooling, the crude product was recrystallized twice from EtOH to produce glutarimide **2** as a white solid (26.22 g, yield 58%) [44]. ^1^H NMR (400 MHz, CDCl_3_): *δ* 1.95–2.02 (m, 2H, CH_2_), 2.57 (t, 4H), 8.49 (s, 1H, NH).

6-Methyl-2-piperidinone (**3**): Into a stirred solution of glutarimide (15.00 g, 0.13 mol) in CH_2_Cl_2_ (100 mL) at −78 °C under a nitrogen atmosphere, the Grignard reagent CH_3_MgBr was added into 2-methyltetrahydrofuran (3 M, 130 mL) dropwise. Then the reaction mixture was stirred at room temperature for 12 h. NaBH_3_CN (9.83 g, 0.16 mol) was added, followed by the slow addition of a 6 N HCl solution to keep the pH at 3–4. The reaction was continued at 25 °C for 5 h. After completion, the mixture was neutralized with 1 N NaOH and extracted with CH_2_Cl_2_ (5 × 100 mL). The combined organic extracts were dried over anhydrous Na_2_SO_4_ and concentrated in vacuo. The crude product was purified by column chromatography on silica gel using ethyl acetate:MeOH (20:1~10:1) to obtain compound **3** as a white solid (9.75 g, yield 65%) [45]. ^1^H NMR (400 MHz, CDCl_3_): *δ* 1.20 (d, 3H, CH_3_), 1.30–1.37 (m, 1H), 1.66–1.74 (m, 1H), 1.87–1.91 (m, 2H), 2.24–2.31 (m, 1H), 2.35–2.41 (m, 1H), 3.48–3.54 (m, 1H), 5.96 (s, br, 1H).

6-Methyl-2-oxo-piperidine-1-carboxylic acid tert-butyl ester (**4**): To a solution of compound **3** (9.75 g, 0.086 mol), Et_3_N (13.07 g, 0.13 mol) and DMAP (0.52 g, 4.3 mmol) in CH_2_Cl_2_ (150 mL), (Boc)_2_O (28.12 g, 0.13 mol) was added. The solution was stirred to reflux for 18 h. When TLC showed that the reaction was complete, the solvent was evaporated and the resulting oil was purified via column chromatography on silica gel using hexanes; ethyl acetate (10:1) was used to obtain compound **4** as a yellow oil (12.86 g, yield 70%) [46]. ^1^H NMR (400 MHz, CDCl_3_): *δ* 1.27 (d, 3H, CH_3_), 1.52 (s, 9H), 1.66–1.77 (m, 2H), 1.90–1.96 (m, 2H), 2.43–2.51 (m, 2H), 4.26–4.31 (m, 1H).

#### 3.1.1. General Procedure for Preparation of N-Boc-aminoketones (**5a**–**c**):

To a stirred solution of tetramethylethylenediamine (TMEDA) (1.5 equiv.) and N-Boc-piperidinone (4) (1 equiv.) in anhydrous THF at −30 °C under a nitrogen atmosphere, the freshly prepared Grignard reagent RMgBr (1.5 equiv.) was added dropwise. The resulting suspension was allowed to be stirred at 25 °C for 10 h. After completion, 1N HCl was added to quench the reaction. The mixture was extracted with CH_2_Cl_2_. The combined organic phase was dried over anhydrous Na_2_SO_4_. The solvent was evaporated and the crude products were isolated by column chromatography on silica gel using hexanes; ethyl acetate (15:1) was used to afford the corresponding compounds.

Tert-butyl 6-oxoheptadecyl-2-carbamate (**5a**): Yield 45% from *n*-C_11_H_23_MgBr, white solid. ^1^H NMR (400 MHz, CDCl_3_): *δ* 0.88 (t, 3H, CH_3_), 1.12 (d, 3H, CH_3_), 1.21–1.31 (m, 16H, CH_2_), 1.37–1.44 (m, 2H, CH_2_), 1.44 (s, 9H, CH_3_ × 3), 1.54–1.61 (m, 4H, CH_2_), 2.36–2.44 (m, 4H, CH_2_), 3.59–3.66 (m, 1H, CH), 4.33 (s, br, 1H, NH); ^13^C NMR (125 MHz, CDCl_3_): *δ* 14.4 (CH_3_), 20.1 (CH_3_), 21.2 (CH_2_), 22.7 (CH_2_), 23.9 (CH_2_), 28.4 (CH_3_ × 3), 29.25 (CH_2_), 29.33 (CH_2_), 29.41 (CH_2_), 29.46 (CH_2_), 29.59 (CH_2_), 31.9 (CH_2_), 36.6 (CH_2_), 42.2 (CH_2_), 42.9 (CH_2_), 46.1 (CH), 79.0 (C), 155.4 (NHCO), 211.2 (CO).

Tert-butyl 6-oxononadecyl -2-carbamate (**5b**): Yield 41% from *n*-C_13_H_27_MgBr, white solid. ^1^H NMR (400 MHz, CDCl_3_): *δ* 0.89 (t, 3H, CH_3_), 1.12 (d, 3H, CH_3_), 1.21–1.32 (m, 22H, CH_2_), 1.44 (s, 9H, CH_3_ × 3), 1.53–1.66 (m, 4H, CH_2_), 2.34–2.55 (m, 4H, CH_2_), 3.59–3.68 (m, 1H, CH), 4.34 (s, br, 1H, NH); ^13^C NMR (125 MHz, CDCl_3_): *δ* 14.1 (CH_3_), 20.1 (CH_3_), 21.2 (CH_2_), 22.7 (CH_2_), 23.9 (CH_2_), 28.4 (CH_3_ × 3), 29.26 (CH_2_), 29.34 (CH_2_), 29.41 (CH_2_), 29.47 (CH_2_), 29.60 (CH_2_), 29.63 (CH_2_), 29.66 (CH_2_), 31.9 (CH_2_), 36.6 (CH_2_), 42.2 (CH_2_), 42.9 (CH_2_), 46.1 (CH), 79.0 (C), 155.4 (NHCO), 211.2 (CO).

Tert-butyl 6-oxohenicosyl -2-carbamate (**5c**): Yield 32% from *n*-C_15_H_27_MgBr, white solid [39]. ^1^H NMR (400 MHz, CDCl_3_): *δ* 0.80 (t, 3H, CH_3_), 1.03 (d, 3H, CH_3_), 1.12–1.26 (m, 26H, CH_2_), 1.35 (s, 9H, CH_3_ × 3), 1.44–1.57 (m, 4H, CH_2_), 2.28–2.35 (m, 4H, CH_2_), 3.52–3.56 (m, 1H, CH), 4.44 (s, br, 1H, NH); ^13^C NMR (125 MHz, CDCl_3_): *δ* 14.0 (CH_3_), 20.0 (CH_3_), 21.1 (CH_2_), 22.6 (CH_2_), 23.4 (CH_2_), 28.3 (CH_3_ × 3), 29.18 (CH_2_), 29.29 (CH_2_), 29.35 (CH_2_), 29.41 (CH_2_), 29.55 (CH_2_), 29.59 (CH_2_), 29.62 (CH_2_), 31.9 (CH_2_), 36.5 (CH_2_), 42.1 (CH_2_), 42.8 (CH_2_), 46.0 (CH), 78.8 (C), 155.4 (NHCO), 211.2 (CO).

#### 3.1.2. General Procedure for Preparation of 2-Methyl-6-alkyl-Δ^1,6^-piperideines (**6a**–**c**)

To a stirred solution of N-Boc-aminoketone (**5a**–**c**) (0.5 g) in CH_2_Cl_2_ (10 mL) at 0 °C, 12 M HCl (5 mL) was added dropwise gradually over 15 min. After stirring for 5 h at 0 °C, the reaction mixture was basified with 2 M NaOH and the resulting mixture was extracted with CH_2_Cl_2_. The combined organic phase was dried over anhydrous Na_2_SO_4_. After workup, the crude product was purified by column chromatography on silica gel using hexanes; ethyl acetate (3:1) was used to produce the 2-methyl-6-alkyl-Δ^1,6^-piperideines.

2-Methyl-6-undecyl-Δ^1,6^-piperideine (**6a**): Yield 85% from **5a** [47]. ^1^H NMR (400 MHz, CDCl_3_): *δ* 0.63 (t, 3H, CH_3_), 0.99 (d, 3H, CH_3_), 1.01–1.05 (m, 16H, CH_2_), 1.27–1.36 (m, 4H, CH_2_), 1.46–1.54 (m, 2H, CH_2_), 1.79–1.98 (m, 4H, CH_2_), 3.21 (m, 1H, CH); ^13^C NMR (125 MHz, CDCl_3_): *δ* 13.8 (CH_3_), 18.2 (CH_3_), 22.5 (CH_2_), 22.9 (CH_2_), 26.8 (CH_2_), 28.2 (CH_2_), 29.01 (CH_2_), 29.13 (CH_2_), 29.25 (CH_2_), 29.33 (CH_2_), 29.40 (CH_2_), 31.7 (CH_2_), 40.7 (CH_2_), 52.6 (CH), 171.3 (C=N); HRMS calculated for C_17_H_33_N [M + H]^+^ = 252.2691, found 252.2685.

2-Methyl-6-tridecyl-Δ^1,6^-piperideine (**6b**): Yield 77% from **5b**. ^1^H NMR (400 MHz, CDCl_3_): *δ* 0.67 (t, 3H, CH_3_), 0.90–1.06 (m, 23H, CH_2_, CH_3_), 1.24–1.38 (m, 4H, CH_2_), 1.51–1.54 (m, 2H, CH_2_), 1.83–1.96 (m, 4H, CH_2_), 3.21 (m, 1H, CH); ^13^C NMR (125 MHz, CDCl_3_): *δ* 13.9 (CH_3_), 18.4 (CH_3_), 22.5 (CH_2_), 23.1 (CH_2_), 26.8 (CH_2_), 28.2 (CH_2_), 29.16 (CH_2_), 29.19 (CH_2_), 29.31 (CH_2_), 29.38 (CH_2_), 29.49 (CH_2_), 29.52 (CH_2_), 31.7 (CH_2_), 41.0 (CH_2_), 52.7 (CH), 170.1 (C=N); HRMS calculated for C_19_H_37_N [M + H]^+^ = 280.3004, found 280.2997.

2-Methyl-6-pentadecyl-Δ^1,6^-piperideine (**6c**): Yield 52% from **5c** [39]. ^1^H NMR (400 MHz, CDCl_3_): *δ* 0.83 (t, 3H, CH_3_), 1.10–1.23 (m, 27H, CH_2_, CH_3_) 1.46–1.55 (m, 4H, CH_2_), 1.67–1.74 (m, 2H, CH_2_), 2.04–2.19 (m, 4H, CH_2_), 3.43 (m, 1H, CH); ^13^C NMR (125 MHz, CDCl_3_): *δ* 14.1 (CH_3_), 18.3 (CH_3_), 22.6 (CH_2_), 23.0 (CH_2_), 27.0 (CH_2_), 28.4 (CH_2_), 29.09 (CH_2_), 29.32 (CH_2_), 29.42 (CH_2_), 29.49 (CH_2_), 29.58 (CH_2_), 29.61 (CH_2_), 29.65 (CH_2_), 31.9 (CH_2_), 40.8 (CH_2_), 52.7 (CH), 171.4 (C=N); HRMS calculated for C_21_H_41_N [M + H]^+^ = 308.3317, found 308.3311.

### 3.2. Comparison of Retention Times and Mass Spectra of Synthetic Δ^1,6^-Piperideines with Those of Corresponding Natural Products

#### 3.2.1. Source of Ant Colony

Workers of the red imported fire ant, *S. invicta*, were collected from Guangzhou, China (113.51° E, 23.18° N). The colony was maintained under laboratory conditions (24–26 °C, relative humidity 60% and 14L:10D h photoperiod) in a 1-gallon plastic jar coated with a Teflon suspension to prevent escape, and was fed sugar water and crickets. Ant workers were subjected to extraction within one month of collection.

#### 3.2.2. Extraction, Isolation and Identification of Δ^1,6^-Piperideines

The extraction and isolation of venom alkaloids from *S. invicta* workers were conducted following previously reported procedures [15,16,48]. Briefly, the hexane extract of ant workers (1 g) randomly selected from the colony, was concentrated to 0.5 mL under a mild stream of nitrogen and loaded onto a 13 mm i.d. gravity chromatography column (20 g of silica gel, 300–400 mesh). The sample was sequentially eluted with hexane and a series of mixtures of hexane and acetone containing 2% triethylamine. Each 2 mL of eluent was collected, and its chemistry was analyzed by a Shimadzu GC–2030 equipped with a flame ionization detector (FID). The dimensions of the capillary column used were as follows: HP–5MS, 30 m × 0.25 mm i.d., 0.25 μm (Agilent). The injector was operated at 250 °C in splitless mode with the split opened after 1 min. Nitrogen was used as the carrier (1 mL/min) and makeup gas. The GC program used was as follows: injection at 90 °C (isothermal for 2 min), increase at 15 °C/min up to 270 °C and hold for 16 min. The detector temperature was set at 280 °C. In terms of appearance of characteristic peaks and their retention times in the GC chromatograms of each collection, the *cis*- and *trans*-alkaloid fractions were obtained by pooling collections containing specific groups of alkaloids. A mixture of synthetic Δ^1,6^-piperideines **6a**, **6b** and **6c** in equal amounts was dissolved in hexane. To confirm the identities of Δ^1,6^-piperideines, both the *cis*-alkaloid fraction with a final volume of 1.5 mL and the solution of synthetic standards were subjected to GC-MS analysis using a Shimadzu GCMS-TQ8050 NX equipped with a Shimadzu SH-Rxi-5Sil MS column with exactly the same dimensions as the HP-5MS column above. The oven temperature was programmed as follows: 90 °C for 1 min, increased at 10 °C/min to 160 °C, then to 250 °C at 3 °C/min, and held for 2 min. The transfer line temperature was set at 250 °C. Mass spectra were obtained using electron impact (EI, 70 eV). Helium was used as the carrier gas (1 mL/min).

### 3.3. Insecticidal Activity Bioassays

#### 3.3.1. Insect Rearing Procedure

Eggs of *Helicoverpa armigera* were purchased from a commercial supplier, Keyun Industry Co., Ltd. (Jiyuan, China). Newly hatched *H. armigera* larvae were reared in groups of 50–100 in 200 mL plastic cups at 27 ± 1 °C, relative humidity 60% and 16L:8D h photoperiod, until the third instar. Small cubes of artificial diet [49] were refreshed daily. The early third-instar larvae were individually transferred to 9 cm-diameter Petri dishes and used for bioassays. The in vivo effect of piperideines **6a**–**c** was determined through topical and injection applications.

#### 3.3.2. Topical Application

Piperideines **6a**–**c** and nicotine were dissolved in acetone and then diluted to obtain the following concentrations: 0.4, 0.2, 0.1 and 0.05 mol/L. Acetone was used as a negative control, whereas nicotine was used as a positive control. A 2 μL drop of acetone solution was applied with a micropipette onto the thoracic dorsum of an anesthetized early third-instar larva placed in a metal bath at 4 °C. Twenty larvae were treated for each treatment. Treated larvae were placed individually in a 10 mL glass tube and maintained on an artificial diet during the post-treatment observation period under the standard rearing conditions describe above. Larvae were considered dead if they were unable to move in a coordinated manner when touched several times with a hairbrush. The numbers of dead larvae were recorded 24 h and 48 h after treatment for the calculation of mortality.

#### 3.3.3. Injection Application

Injections of **6a**–**c** and nicotine solutions at 0.4 mol/L were performed using the Nanoject II Injector (Drummond Scientific Company, Broomall, PA, USA). The needle of the injector was carefully inserted between the second and third abdominal segments of an anesthetized third-instar larva placed in the icy metal bath at a vertical angle of about 30° under a dissecting microscope. A total of 202.4 nL (50.6 nL × 4) of the sample was applied to a single larva. Sixteen larvae were treated in each treatment. The tests were replicated five times over 5 consecutive days. Acetone was served as the control. The treated larvae were maintained separately under standard conditions. Mortality was recorded 24 h and 48 h after treatment.

## 4. Conclusions

In summary, three minor components of the fire ant venom and 2-methyl-6-alkyl-Δ^1,6^-piperideines were synthesized and used to confirm the identification of the corresponding natural products. Their retention times and mass spectra data coincided with those in the *cis*-alkaloid fraction purified from the hexane extract of *S. invicta* workers. Their insecticidal activities were further evaluated with topical application and injection methods. The results of the bioassays indicated that these compounds displayed insecticidal properties against the early third-instar larvae of *H. armigera*. In particular, compound **6a,** with an undecyl side chain, displayed promising injection activity as compared to the natural alkaloid nicotine at 0.4 mol/L. The activity of 2-methyl-6-alkyl-Δ^1,6^-piperideines is negatively correlated to the length of the carbon chain at the 6-position of the piperideine ring. Interestingly, the piperideines were found to be almost inactive when applied topically. The cuticle of the cotton bollworm may prevent the penetration of piperideines into the haemolymph, resulting in such inactivity by topical application. The insecticidal activities of compounds **6a**–**c** provide valuable information for further research on biological activities.

## Figures and Tables

**Table 1:** Contact toxicities of **6a**–**c** against the early third-instar larvae of cotton bollworm following topical application.

Compound	Concentration (mol/L)	Mortality (%)	
		24 h	48 h
**6a**	0.4	15.0	15.0
	0.2	5.0	15.0
	0.1	0	0
	0.05	0	0
**6b**	0.4	5.0	5.0
	0.2	0	0
	0.1	0	0
	0.05	0	0
**6c**	0.4	0	0
	0.2	0	5
	0.1	0	0
	0.05	0	0
Nicotine	0.4	5.0	10.0
	0.2	0	0
	0.1	0	0
	0.05	0	0
Control (Acetone)	-	0	0

**Table 2:** Insecticidal activities of **6a**–**c** against the early third-instar larvae of cotton bollworm following injection application.

Compound	Mortality (%) (Mean ± SD) ^A^	
	24 h	48 h
**6a ^B^**	45.5 ± 15.9 d	75.6 ± 5.7 e
**6b ^B^**	27.6 ± 12.8 bc	48.5 ± 24.9 d
**6c ^B^**	20.1 ± 10.1 b	37.2 ± 2.4 cd
Nicotine ^B^	32.9 ± 10.2 bcd	43.7 ± 6.0 d
Acetone	1.3 ± 2.8 a	2.5 ± 3.4 a

^A^ The means followed by different letters indicate significant difference (*p* < 0.05) in same column by the Tukey-HSD test. ^B^ The concentration of test compounds and nicotine is 0.4 mol/L.

## Data Availability

Not applicable.

## Supplementary Materials

The following supporting information can be downloaded online: Figures S1–S3: ^1^H NMR of **2**–**4**, Figures S4–S9: ^1^H NMR and ^13^C NMR of **5a**–**c**, Figures S10–S18: ^1^H NMR, ^13^C NMR, and HRMS of **6a**–**c**.

## Sample Availability

Samples of the compounds are available from the authors.
