# Supplementary material for: Synthesis and Insecticidal Activity of Fire Ant Venom Alkaloid-Based 2-Methyl-6-alkyl-Δ1,6-piperideines
Source: Molecules. 2022 Feb 7;27(3):1107. doi: 10.3390/molecules27031107 (PMC8838720; doi:10.3390/molecules27031107)
Supplement: Supplementary file 1 [file molecules-27-01107-s001.zip › molecules-1545499-supplementary.pdf]

Synthesis and insecticidal activity of Fire Ant Venom Alkaloid Based 2-Methyl-6-alkyl-$\Delta^{1,6}$-piperideines

Table of Contents

S1. ^{1}H NMR Spectrum of Compound **2**
S2. ^{1}H NMR Spectrum of Compound **3**
S3. ^{1}H NMR Spectrum of Compound **4**
S4. ^{1}H NMR Spectrum of Compound **5a**
S5. ^{13}C NMR Spectrum of Compound **5a**
S6. ^{1}H NMR Spectrum of Compound **5b**
S7. ^{13}C NMR Spectrum of Compound **5b**
S8. ^{1}H NMR Spectrum of Compound **5c**
S9. ^{13}C NMR Spectrum of Compound **5c**
S10. ^{1}H NMR Spectrum of Compound **6a**
S11. ^{13}C NMR Spectrum of Compound**6a**
S12. HRMS Spectrum of Compound **6a**
S13. ^{1}H NMR Spectrum of Compound **6b**
S14. ^{13}C NMR Spectrum of Compound**6b**
S15. HRMS Spectrum of Compound **6b**
S16. ^{1}H NMR Spectrum of Compound **6c**
S17. ^{13}C NMR Spectrum of Compound**6c**
S18. HRMS Spectrum of Compound **6c**

S1. ^{1}H NMR Spectrum of Compound 2

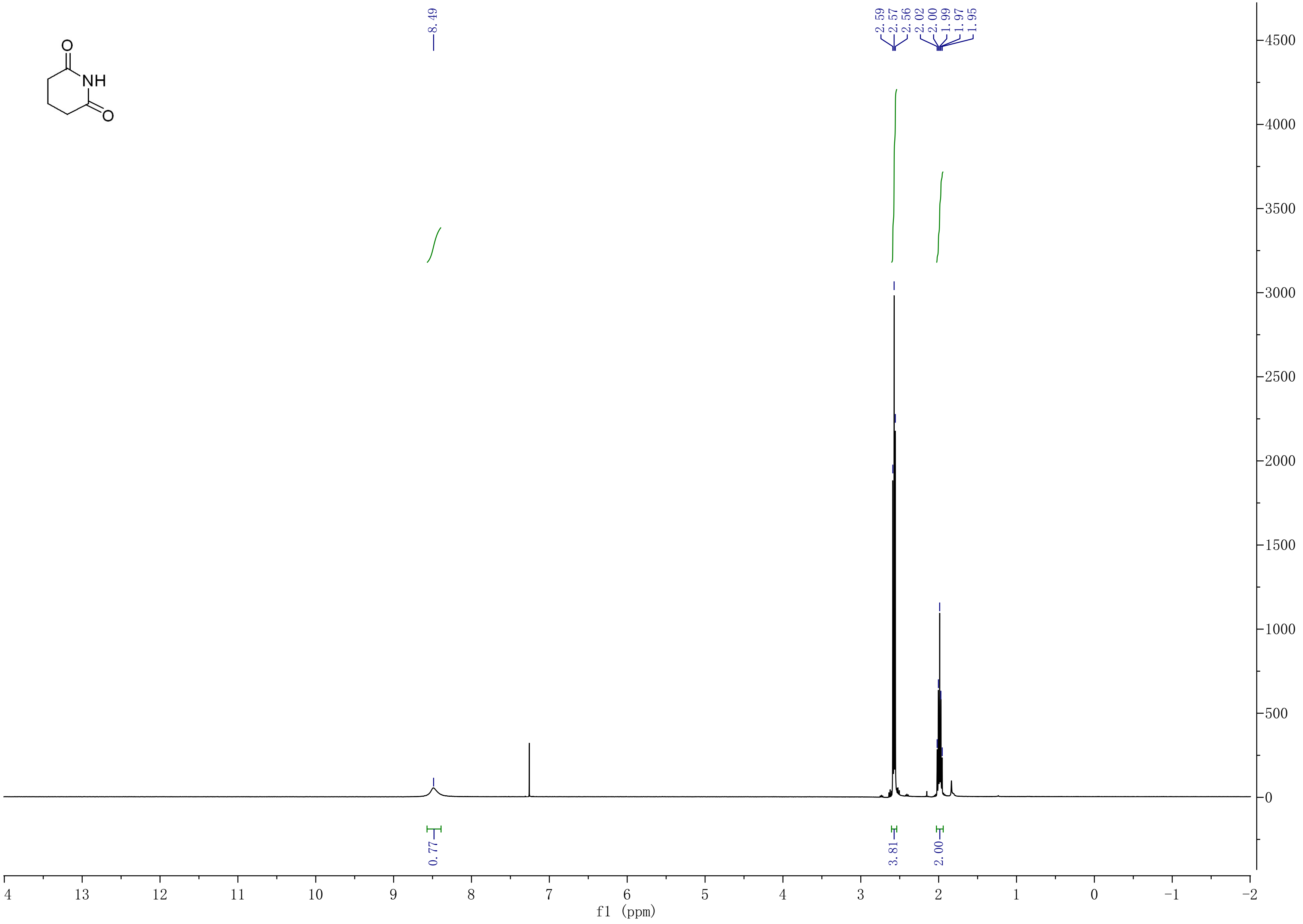

S2. ^{1}H NMR Spectrum of Compound 3

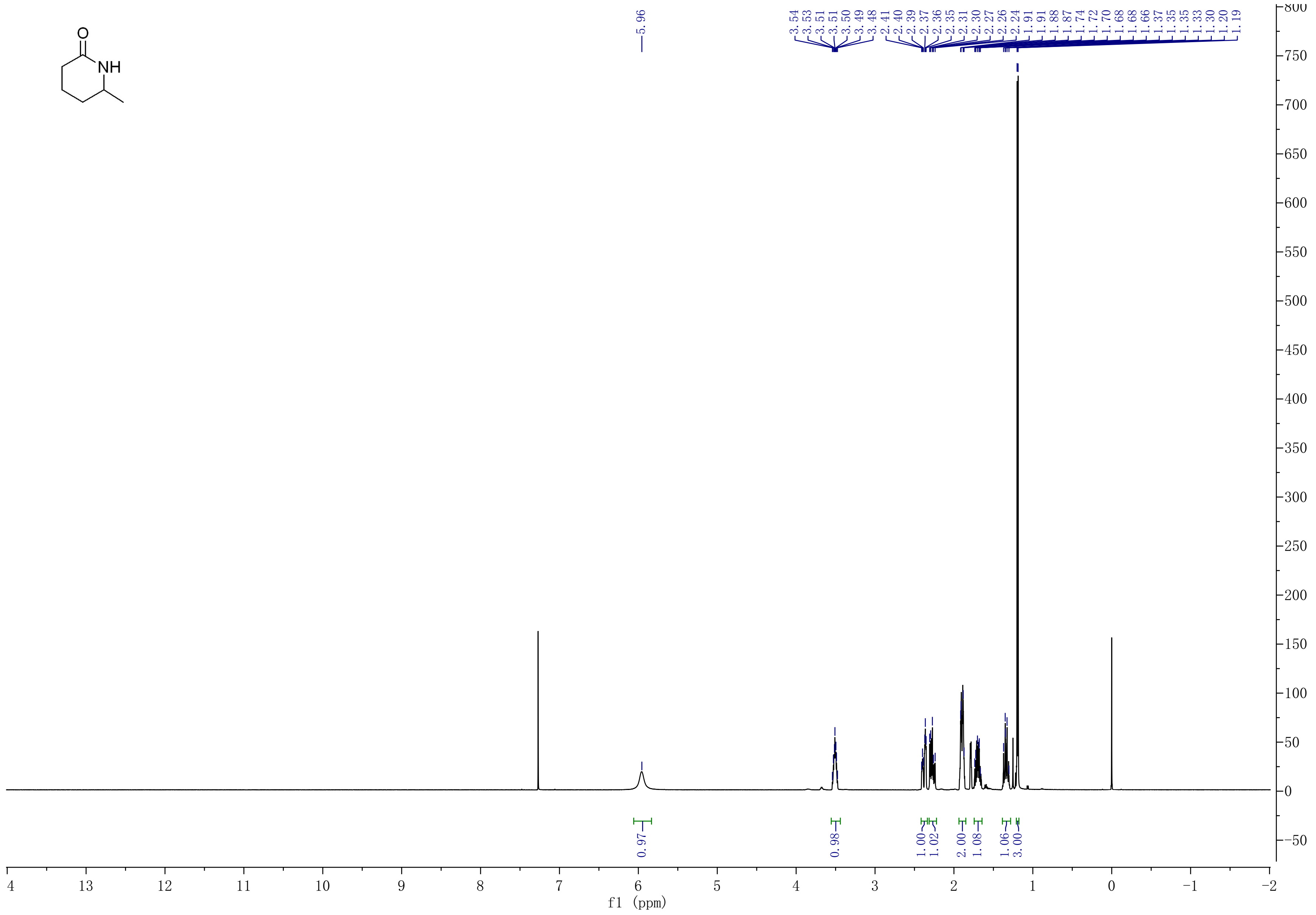

S3. ^{1}H NMR Spectrum of Compound 4

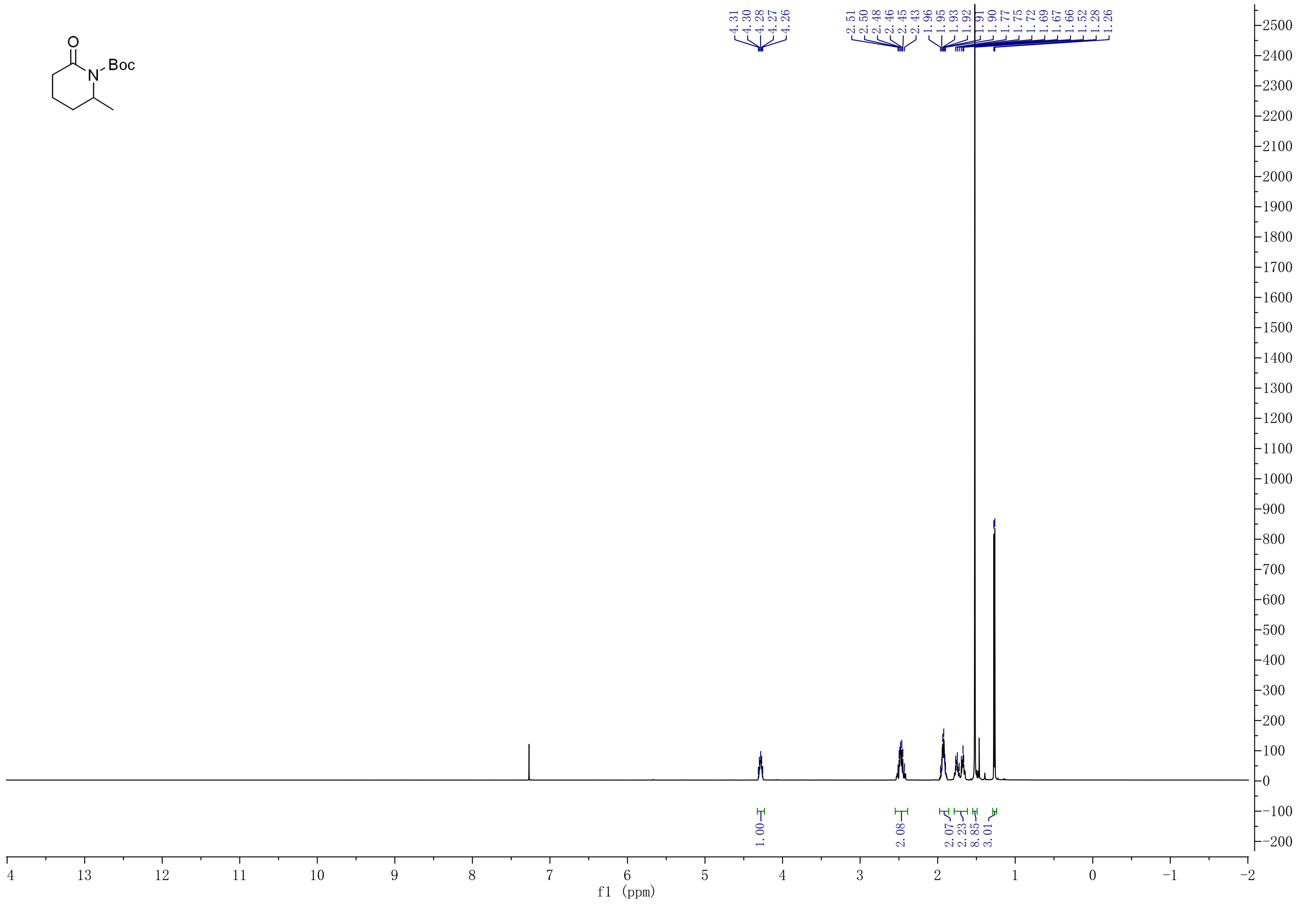

S4. ¹H NMR Spectrum of Compound 5a

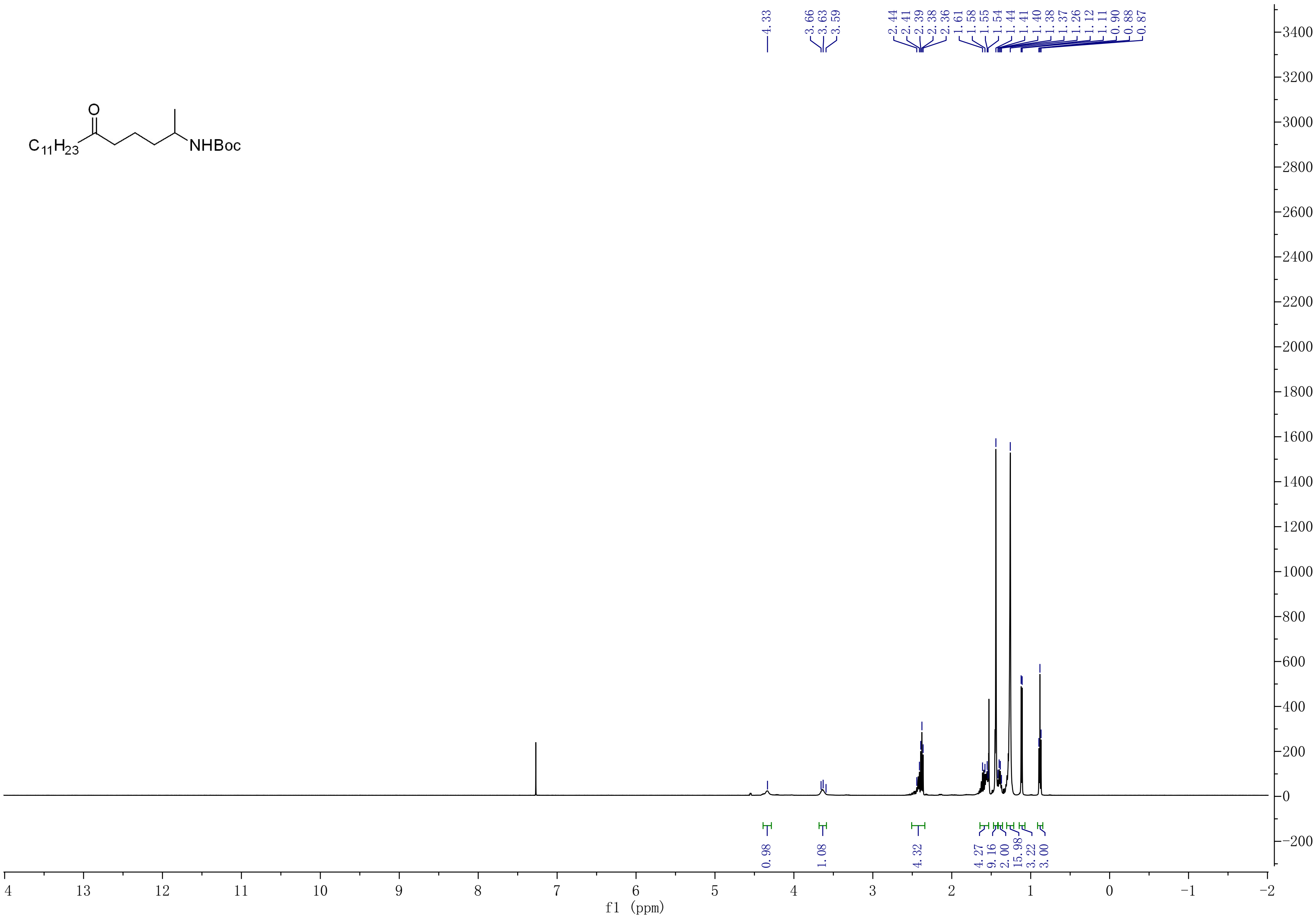

S5. ^{13}C NMR Spectrum of Compound 5a

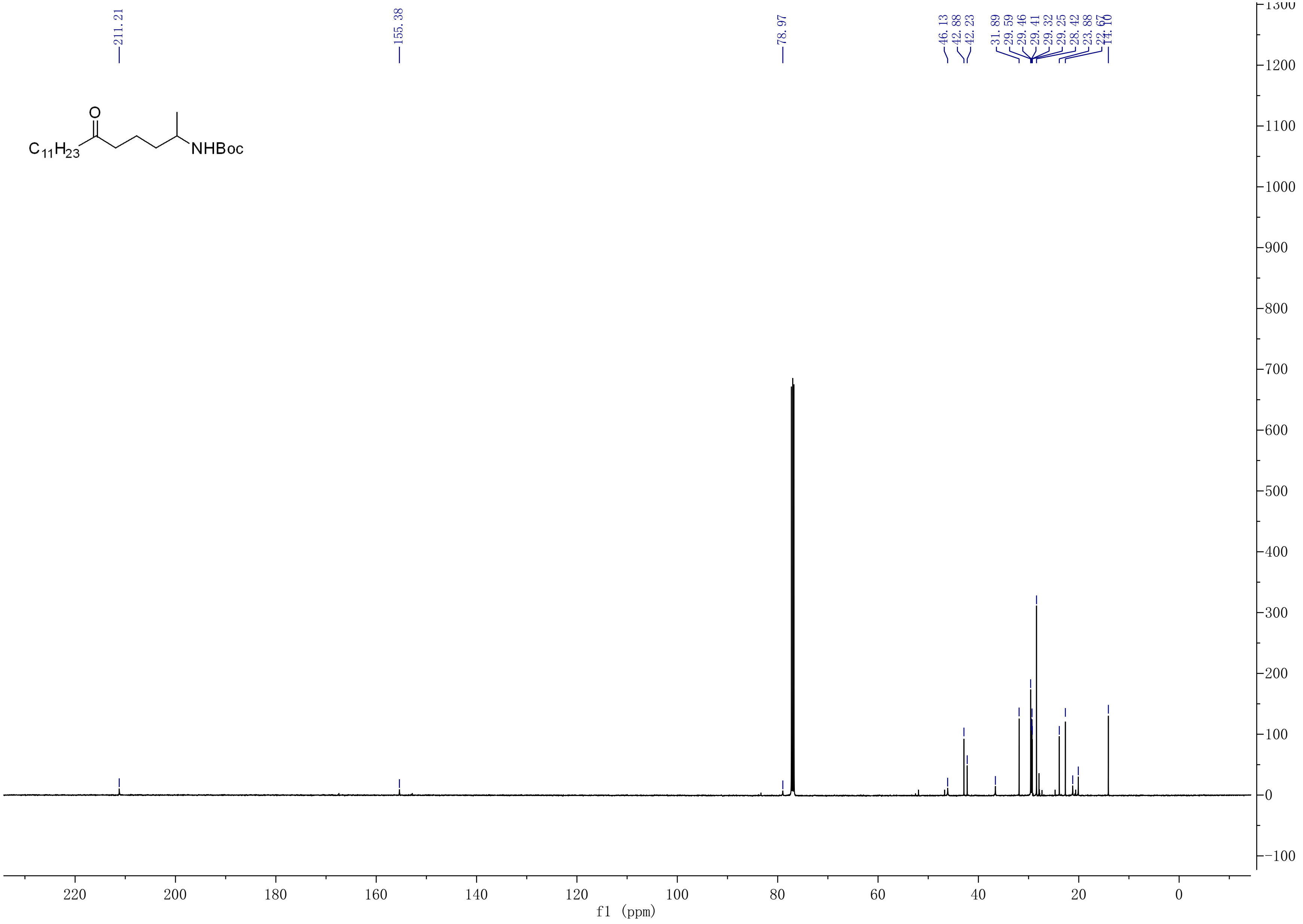

S6. ^{1}H NMR Spectrum of Compound 5b

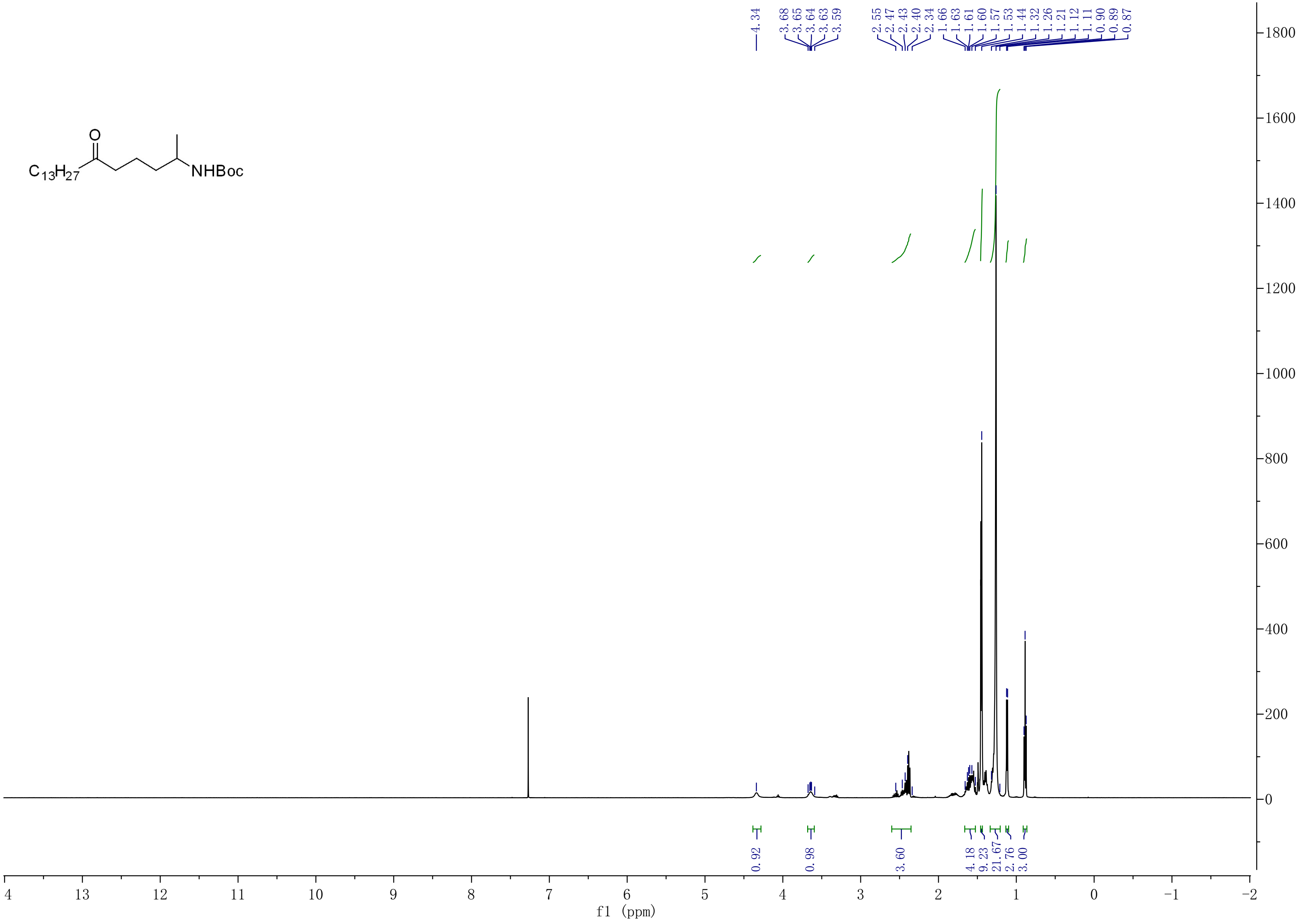

S7. ^{13}C NMR Spectrum of Compound 5b

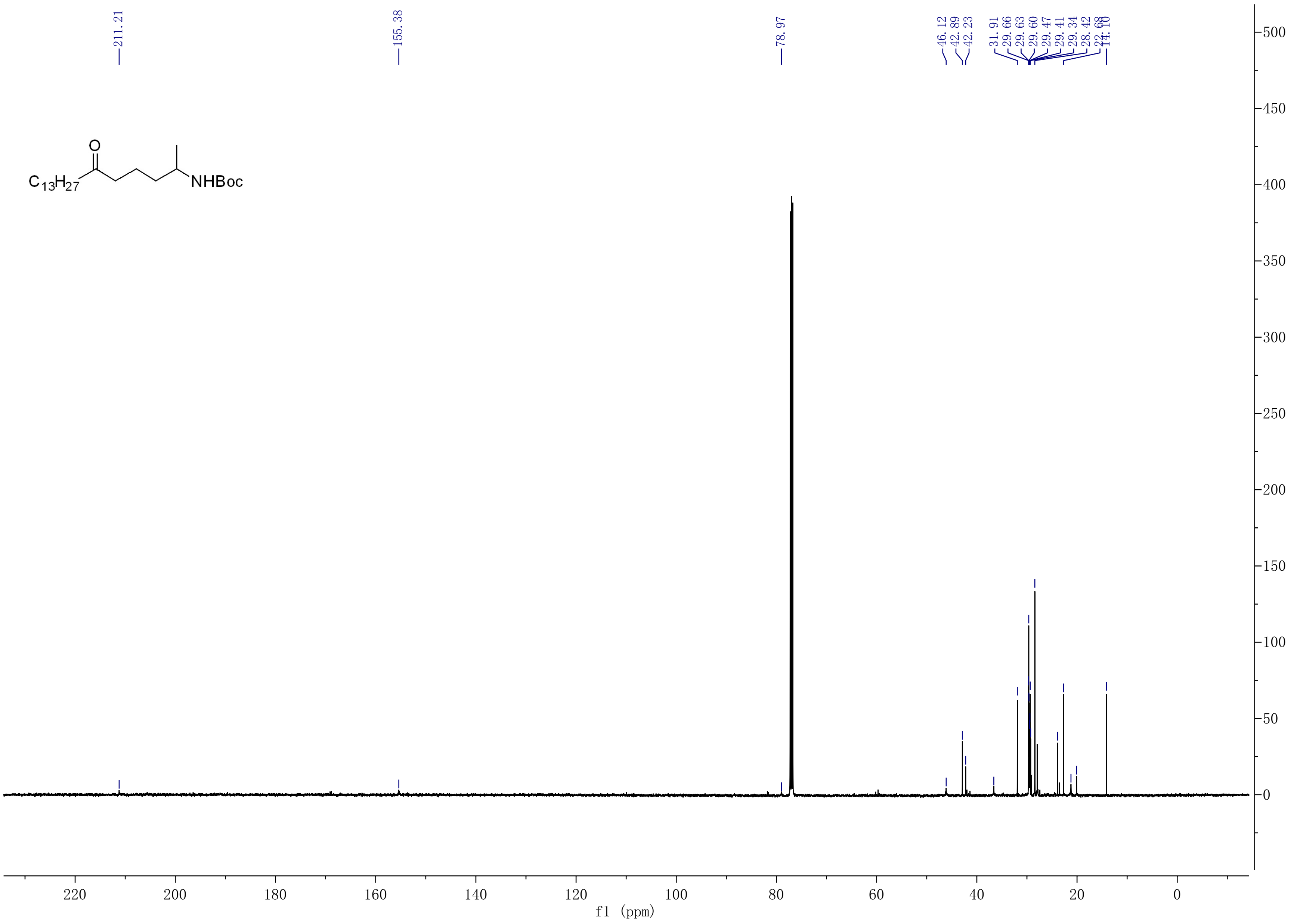

S8. 1H NMR Spectrum of Compound 5c

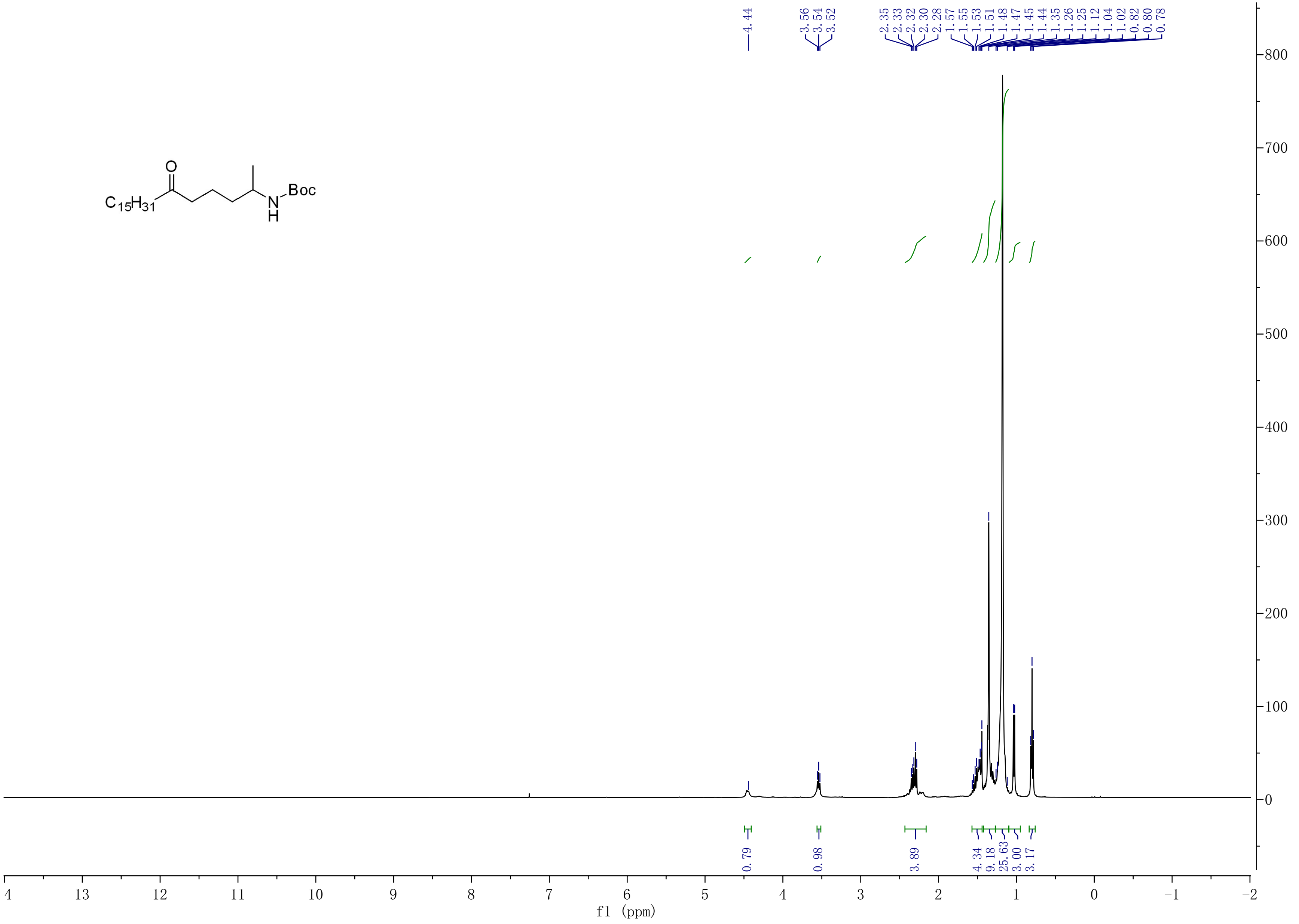

S9. ^{13}C NMR Spectrum of Compound 5c

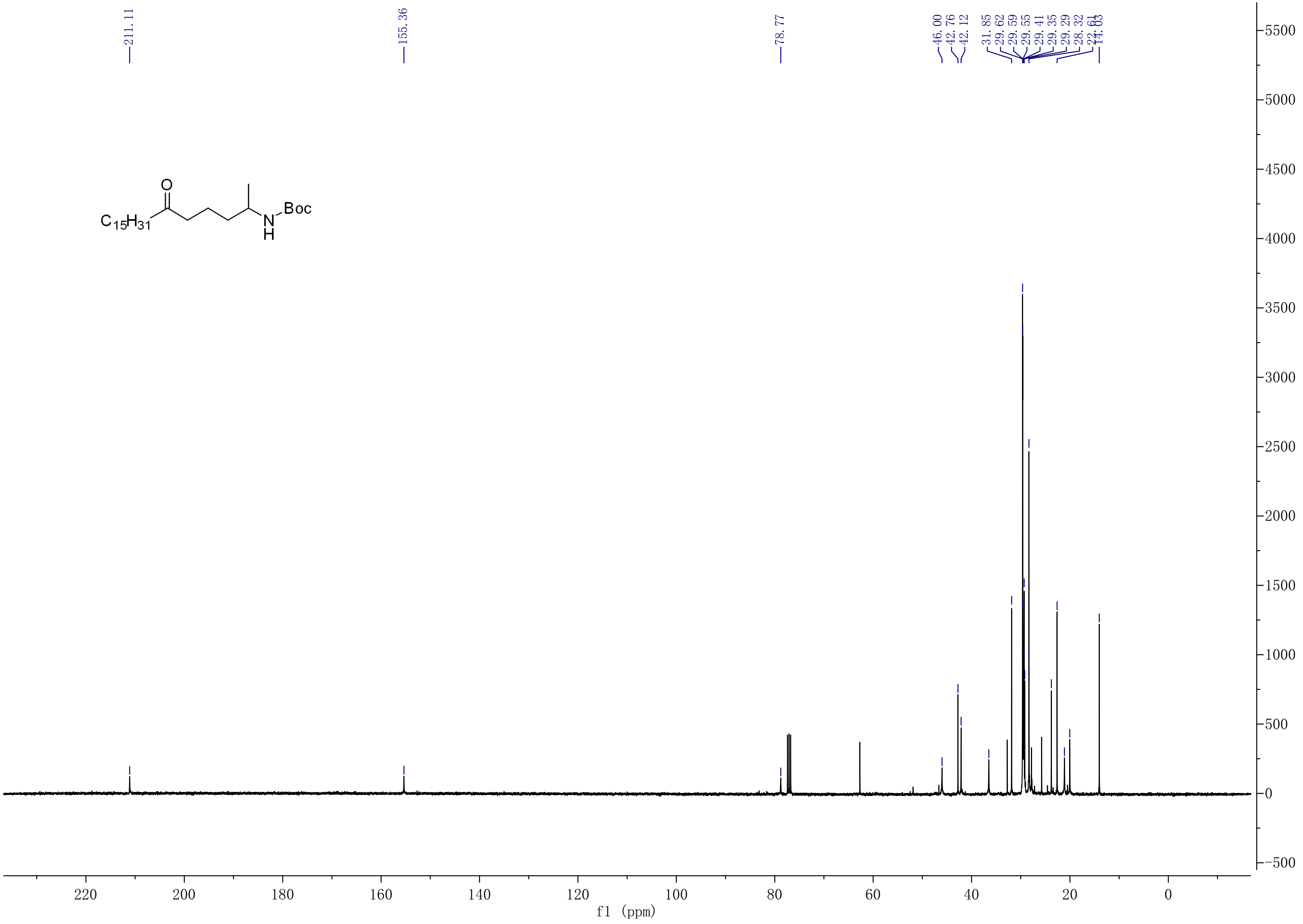

S10. ^{1}H NMR Spectrum of Compound 6a

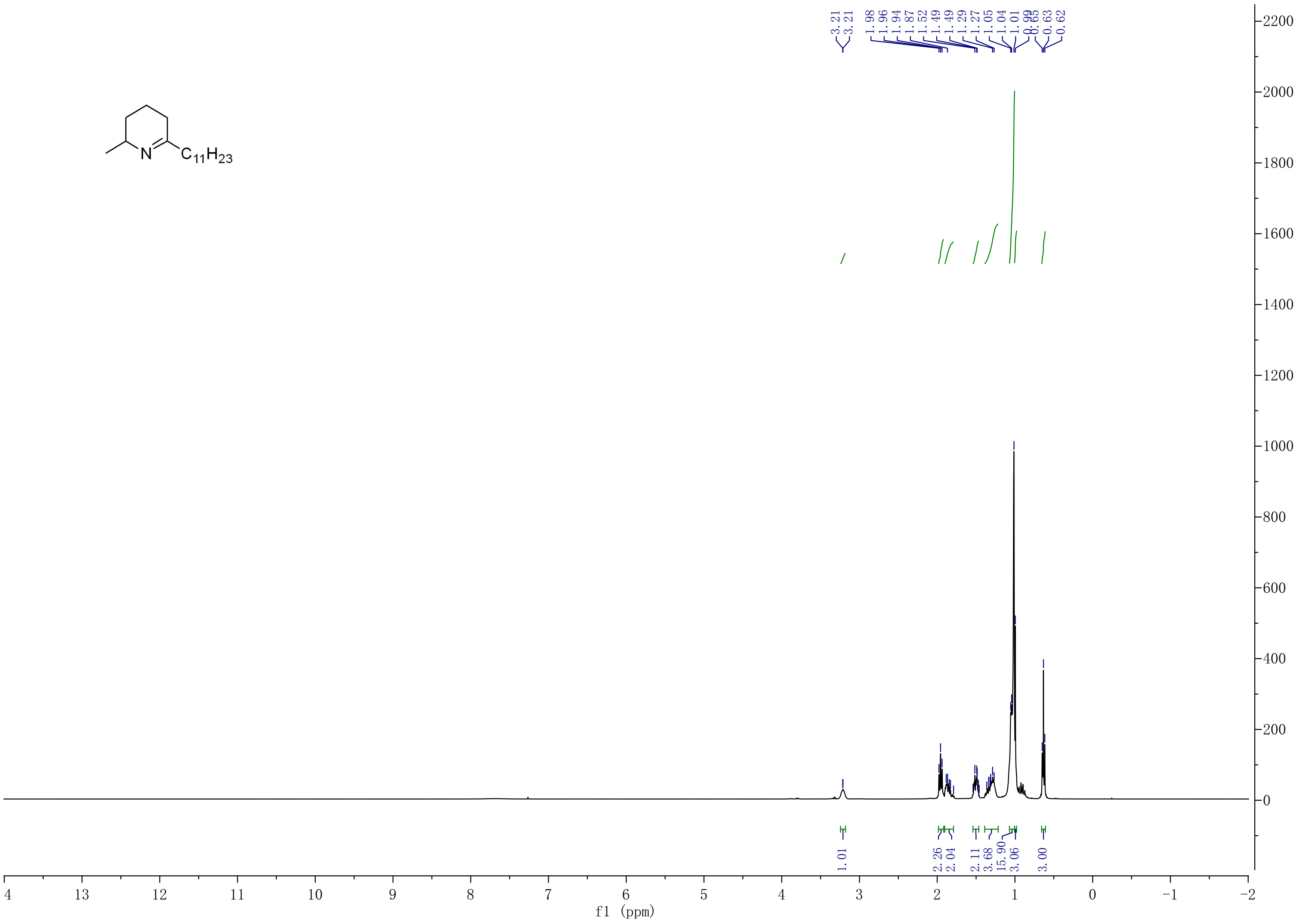

S11. ^{13}C NMR Spectrum of Compound6a

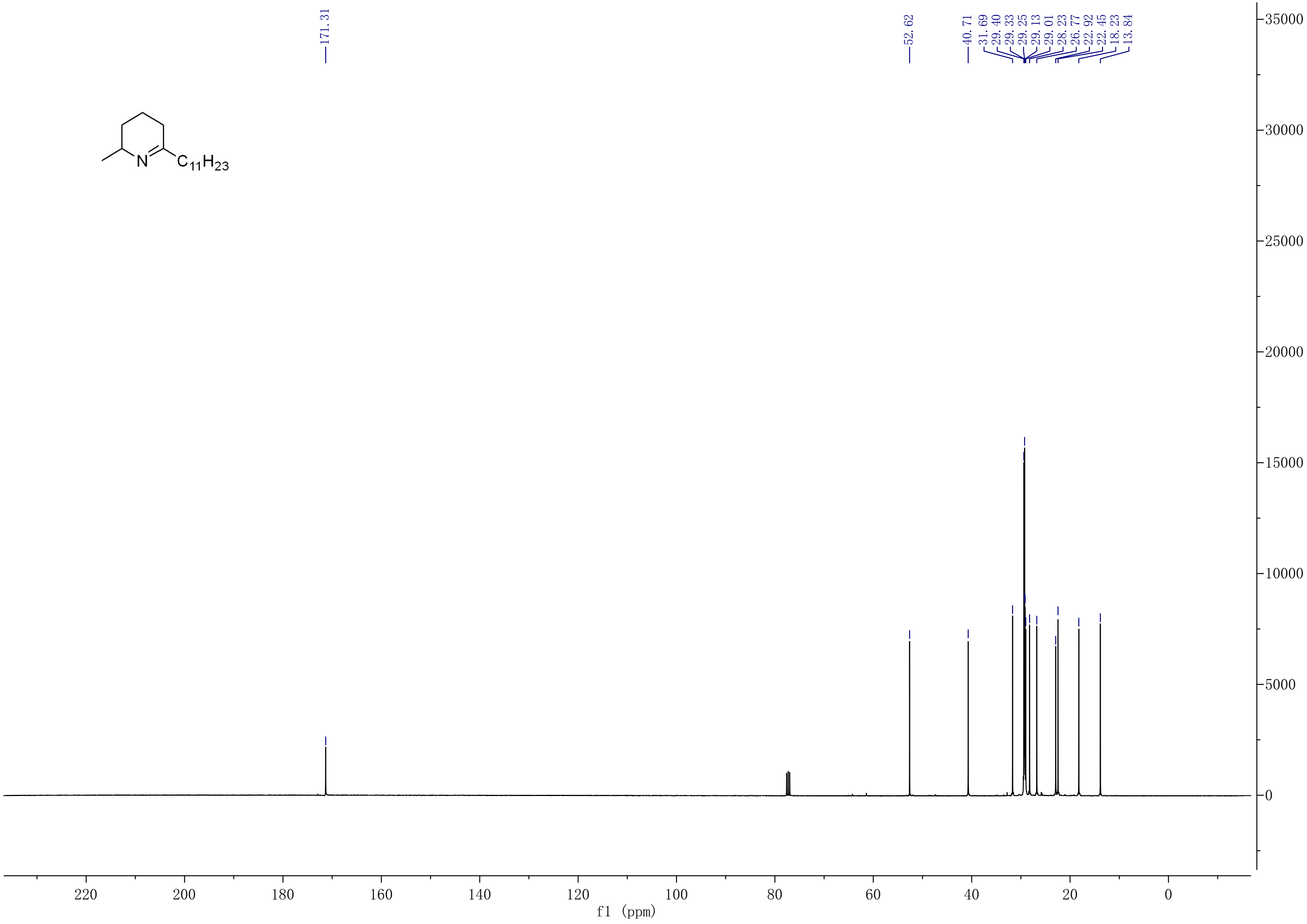

S12. HRMS Spectrum of Compound 6a

Mass Spectrum SmartFormula Report

Analysis Info

Analysis Name	D:\Data\2020.09.25 chen li\pos-1_000001.d	Acquisition Date	9/25/2020 5:32:03 PM
Method	ESI_101506	Operator	
Sample Name	TUNMIX	Instrument	apex-Ultra
Comment			

Acquisition Parameter

Polarity	Positive	Source	ESI	No. of Laser Shots	20
Averaged Scans	16	No. of Cell Fills	1	Laser Power	51.0 %
Broadband Low Mass	100.3 m/z	End Plate	4000.0 V	MALDI Plate	300.0 V
Broadband High Mass	2500.0 m/z	Capillary Entrance	4500.0 V	Imaging Spot Diameter	2000.0 µm
Acquisition Mode	Single MS	Skimmer 1	30.0 V		
Pulse Program	basic	Drying Gas Temperature	180.0 °C	Calibration Date	Fri Sep 25 08:42:02 2020
Source Accumulation	0.0 sec	Drying Gas Flow Rate	4.0 L/min	Data Acquisition Size	524288
Ion Accumulation Time	1.0 sec	Nebulizer Gas Flow Rate	1.0 L/min	Apodization	Sine-Bell Multiplication
Flight Time to Acq. Cell	0.0 sec				

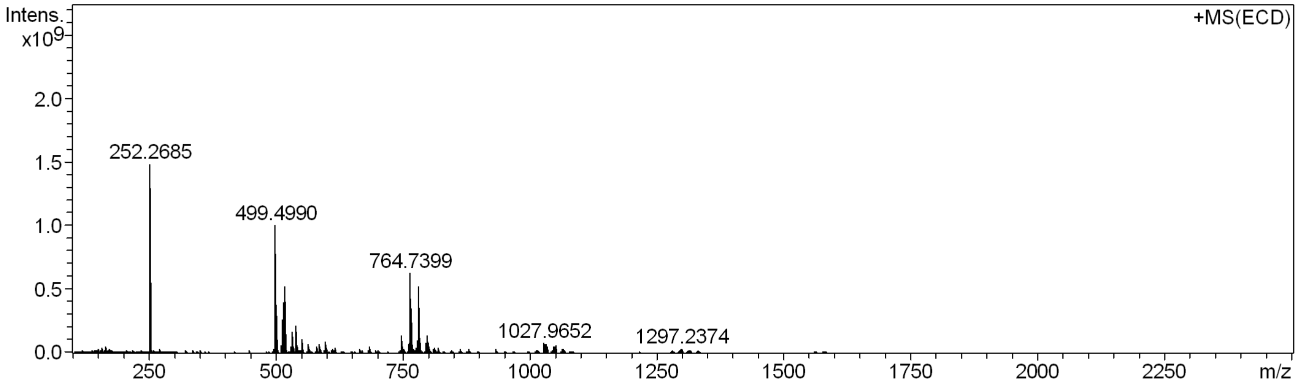

Meas. m/z	#	Formula	Score	m/z	err [mDa]	err [ppm]	mSigma	rdb	e⁻ Conf	N-Rule

S13. ^{1}H NMR Spectrum of Compound 6b

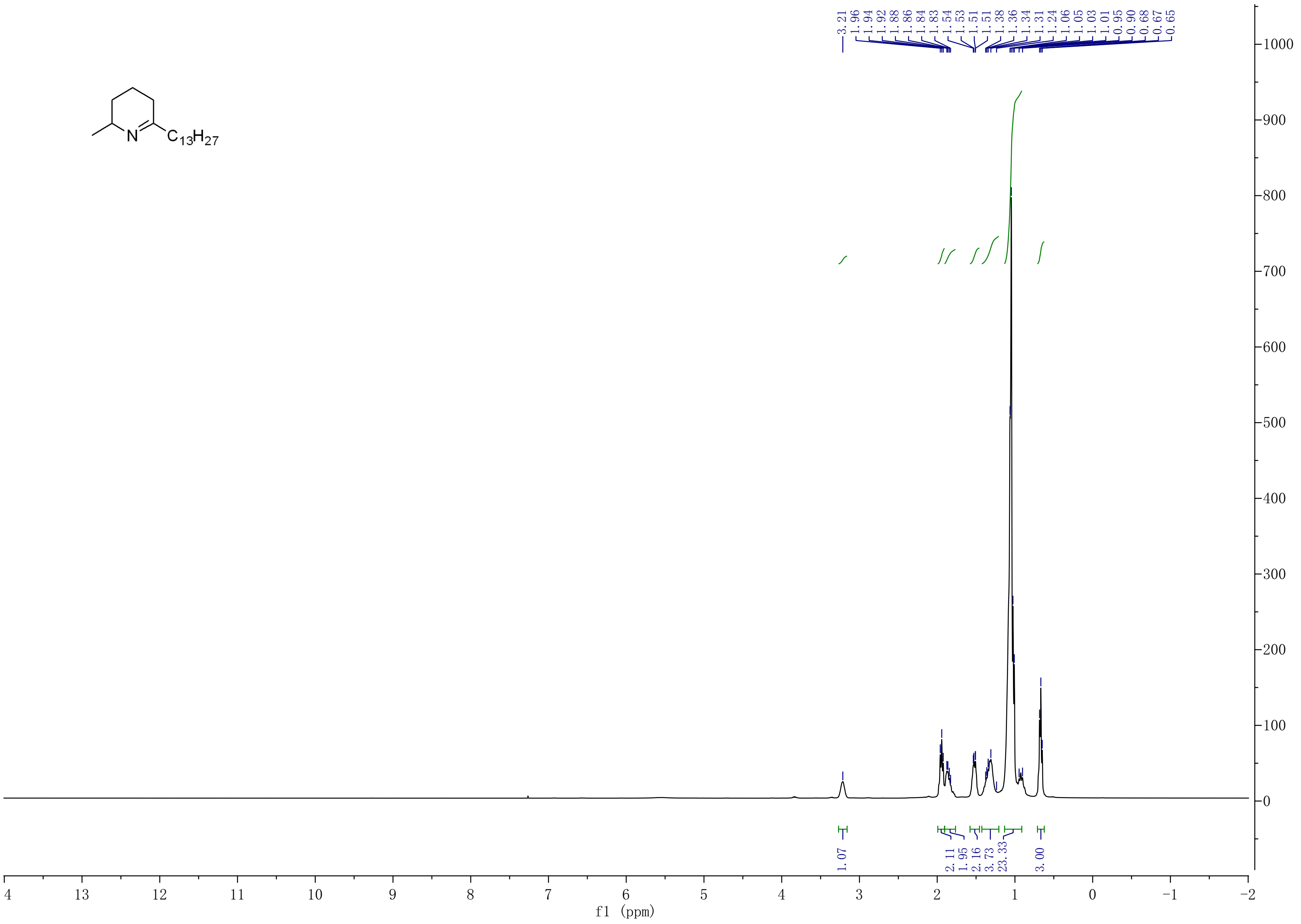

S14. ^{13}C NMR Spectrum of Compound6b

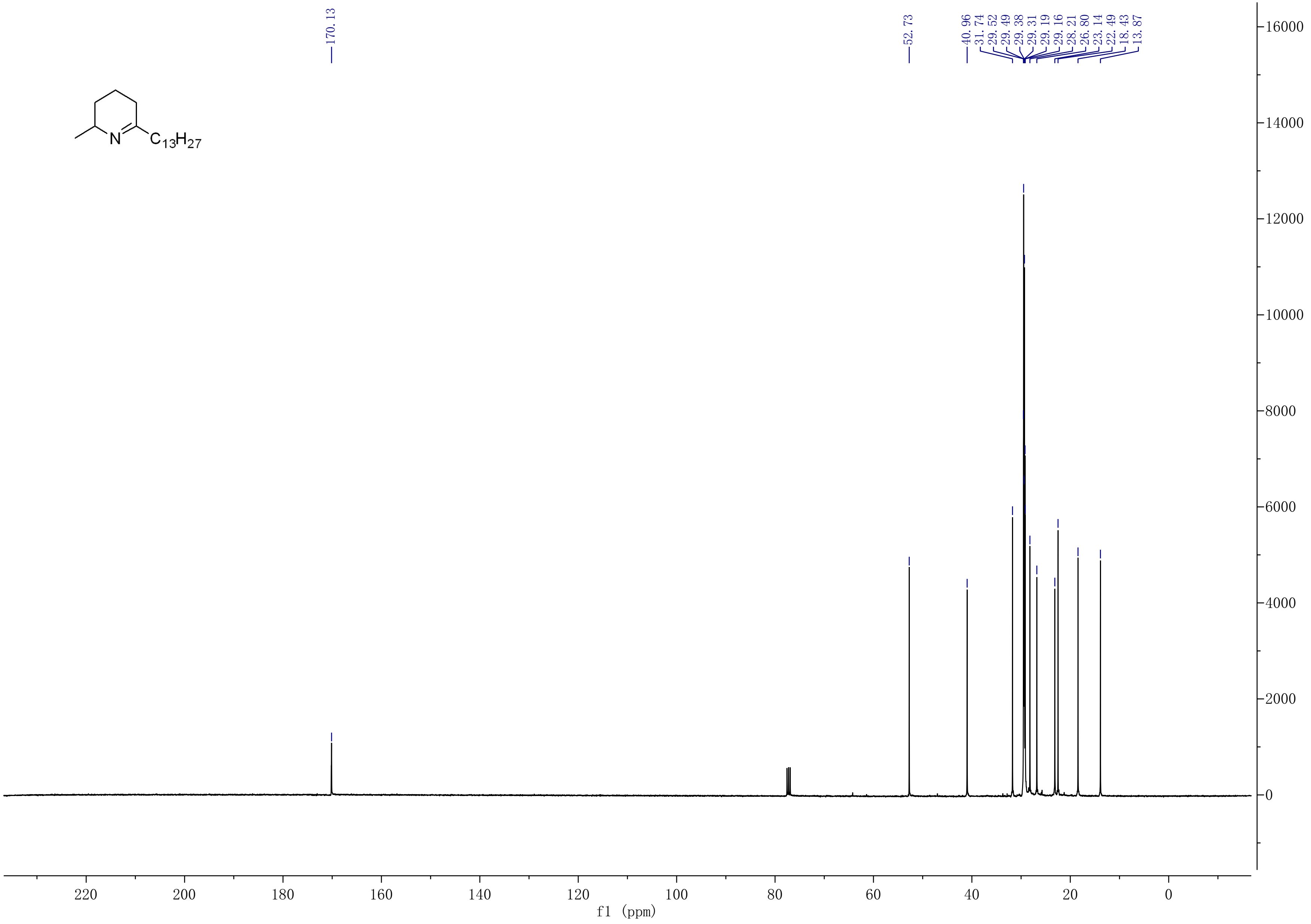

S15. HRMS Spectrum of Compound 6b

Mass Spectrum SmartFormula Report

Analysis Info

Analysis Name	D:\Data\2020.09.25 chen li\pos-2_000001.d	Acquisition Date	9/25/2020 5:47:23 PM
Method	ESI_101506	Operator	
Sample Name	TUNMIX	Instrument	apex-Ultra
Comment			

Acquisition Parameter

Polarity	Positive	Source	ESI	No. of Laser Shots	20
Averaged Scans	16	No. of Cell Fills	1	Laser Power	51.0 %
Broadband Low Mass	100.3 m/z	End Plate	4000.0 V	MALDI Plate	300.0 V
Broadband High Mass	2500.0 m/z	Capillary Entrance	4500.0 V	Imaging Spot Diameter	2000.0 µm
Acquisition Mode	Single MS	Skimmer 1	30.0 V		
Pulse Program	basic	Drying Gas Temperature	180.0 °C	Calibration Date	Fri Sep 25 08:42:02 2020
Source Accumulation	0.0 sec	Drying Gas Flow Rate	4.0 L/min	Data Acquisition Size	524288
Ion Accumulation Time	1.0 sec	Nebulizer Gas Flow Rate	1.0 L/min	Apodization	Sine-Bell Multiplication
Flight Time to Acq. Cell	0.0 sec				

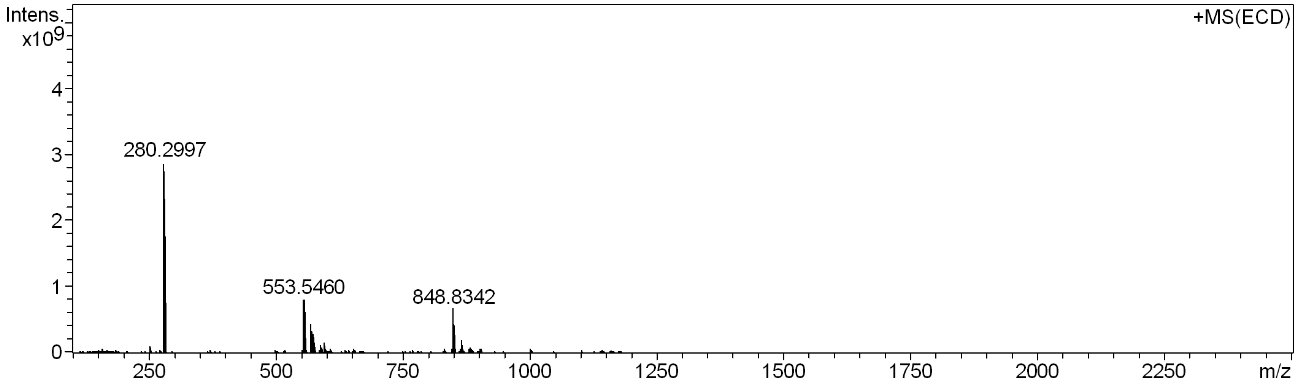

Meas. m/z	#	Formula	Score	m/z	err [mDa]	err [ppm]	mSigma	rdb	e⁻ Conf	N-Rule

S16. ^{1}H NMR Spectrum of Compound 6c

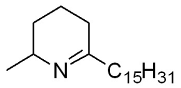

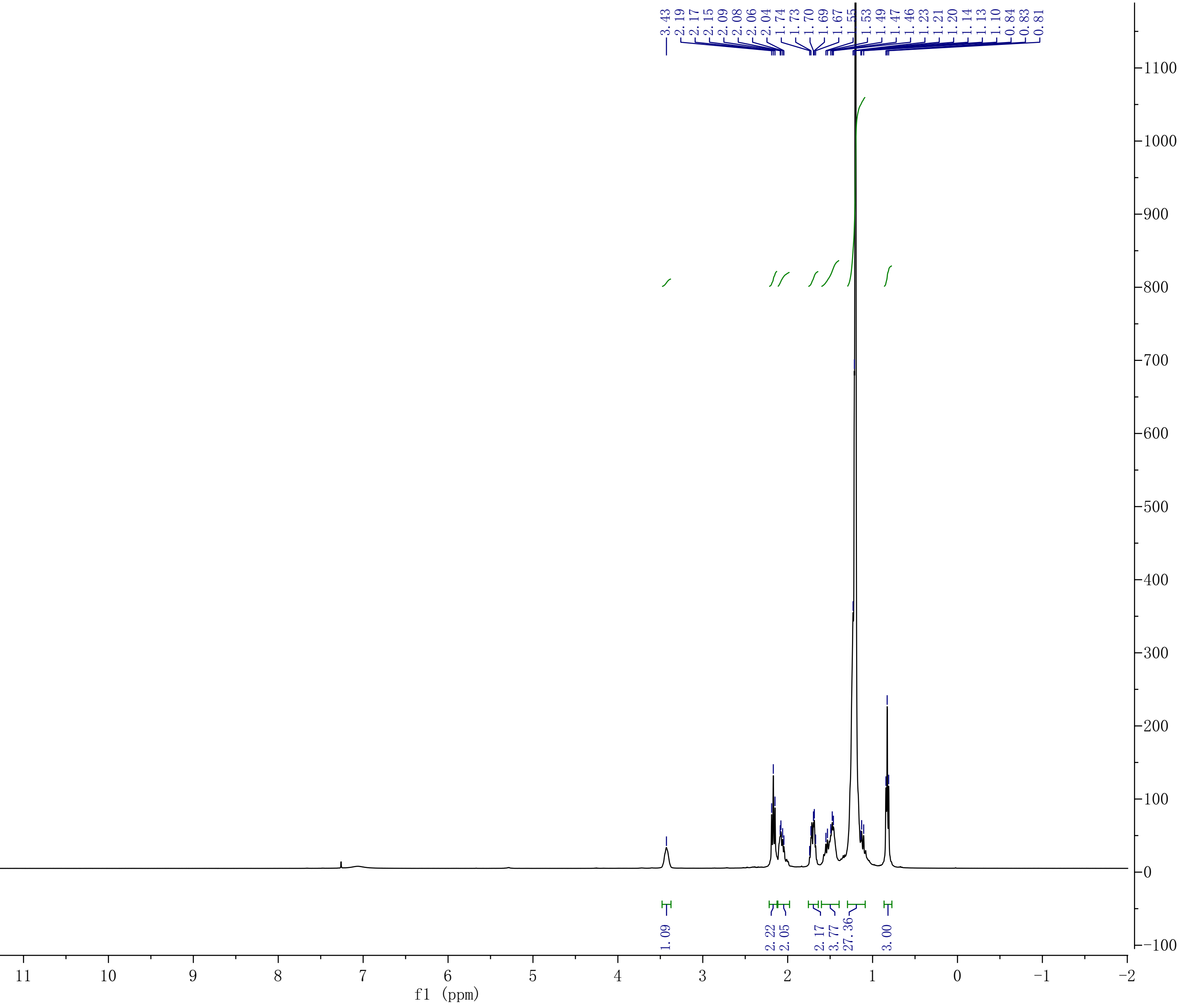

S17. ^{13}C NMR Spectrum of Compound6c

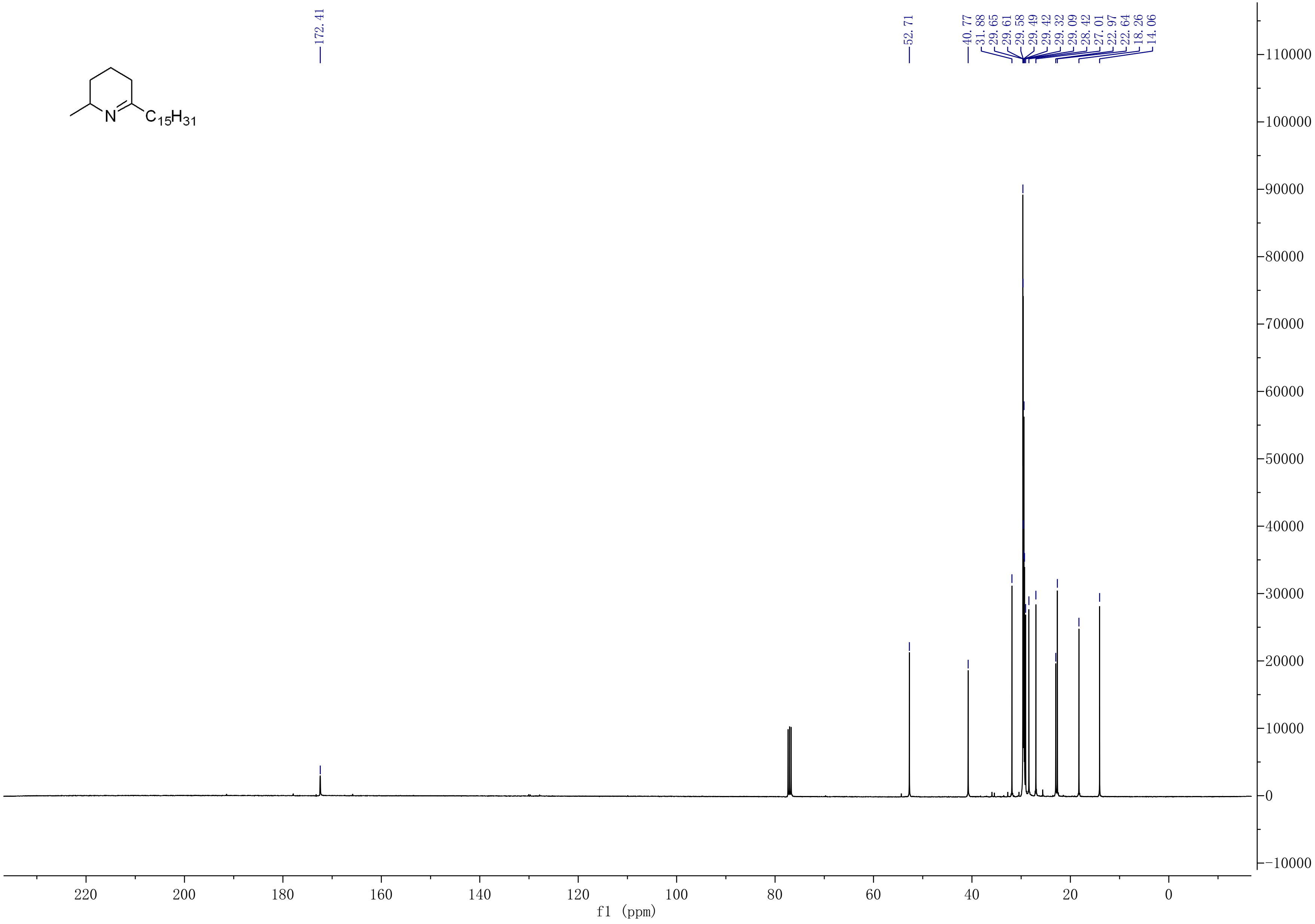

S18. HRMS Spectrum of Compound 6c

Mass Spectrum SmartFormula Report

Analysis Info

Analysis Name	D:\Data\2020.09.25 chen li\pos-3_000001.d	Acquisition Date	9/25/2020 5:51:47 PM
Method	ESI_101506	Operator	
Sample Name	TUNMIX	Instrument	apex-Ultra
Comment			

Acquisition Parameter

Polarity	Positive	Source	ESI	No. of Laser Shots	20
Averaged Scans	16	No. of Cell Fills	1	Laser Power	51.0 %
Broadband Low Mass	100.3 m/z	End Plate	4000.0 V	MALDI Plate	300.0 V
Broadband High Mass	2500.0 m/z	Capillary Entrance	4500.0 V	Imaging Spot Diameter	2000.0 µm
Acquisition Mode	Single MS	Skimmer 1	30.0 V		
Pulse Program	basic	Drying Gas Temperature	180.0 °C	Calibration Date	Fri Sep 25 08:42:02 2020
Source Accumulation	0.0 sec	Drying Gas Flow Rate	4.0 L/min	Data Acquisition Size	524288
Ion Accumulation Time	1.0 sec	Nebulizer Gas Flow Rate	1.0 L/min	Apodization	Sine-Bell Multiplication
Flight Time to Acq. Cell	0.0 sec				

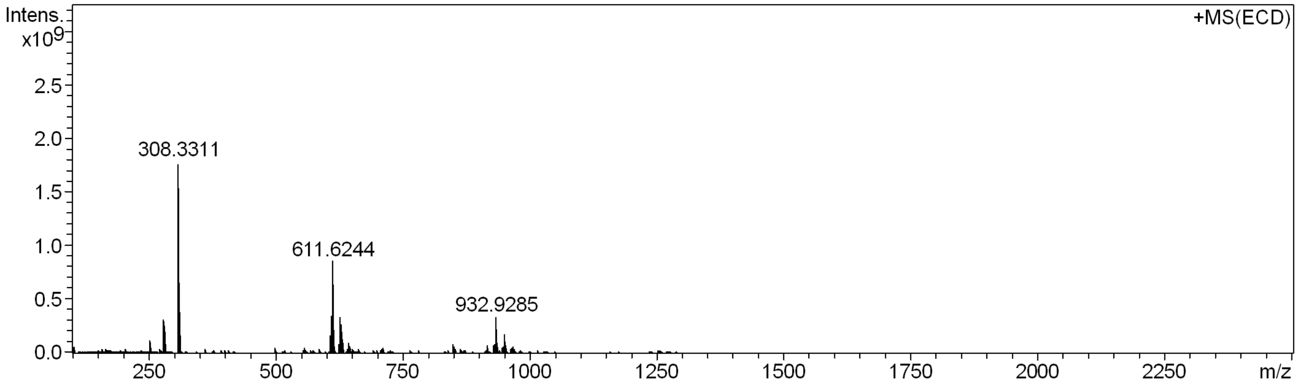

Meas. m/z	#	Formula	Score	m/z	err [mDa]	err [ppm]	mSigma	rdb	e⁻ Conf	N-Rule
